# Supplementary material for: Combined aptamer and transcriptome sequencing of single cells
Source: Sci Rep. 2018 Feb 13;8:2919. doi: 10.1038/s41598-018-21153-y (PMC5811598; doi:10.1038/s41598-018-21153-y)
Supplement: Supplementary file 1 — Supplementary Information [file 41598_2018_21153_MOESM1_ESM.pdf]

Supplementary Information: Combined aptamer and transcriptome sequencing of single cells

Cyrille L. Delley, Leqian Liu, Maen F. Sarhan & Adam R. Abate

Description: Supplementary tables and figures

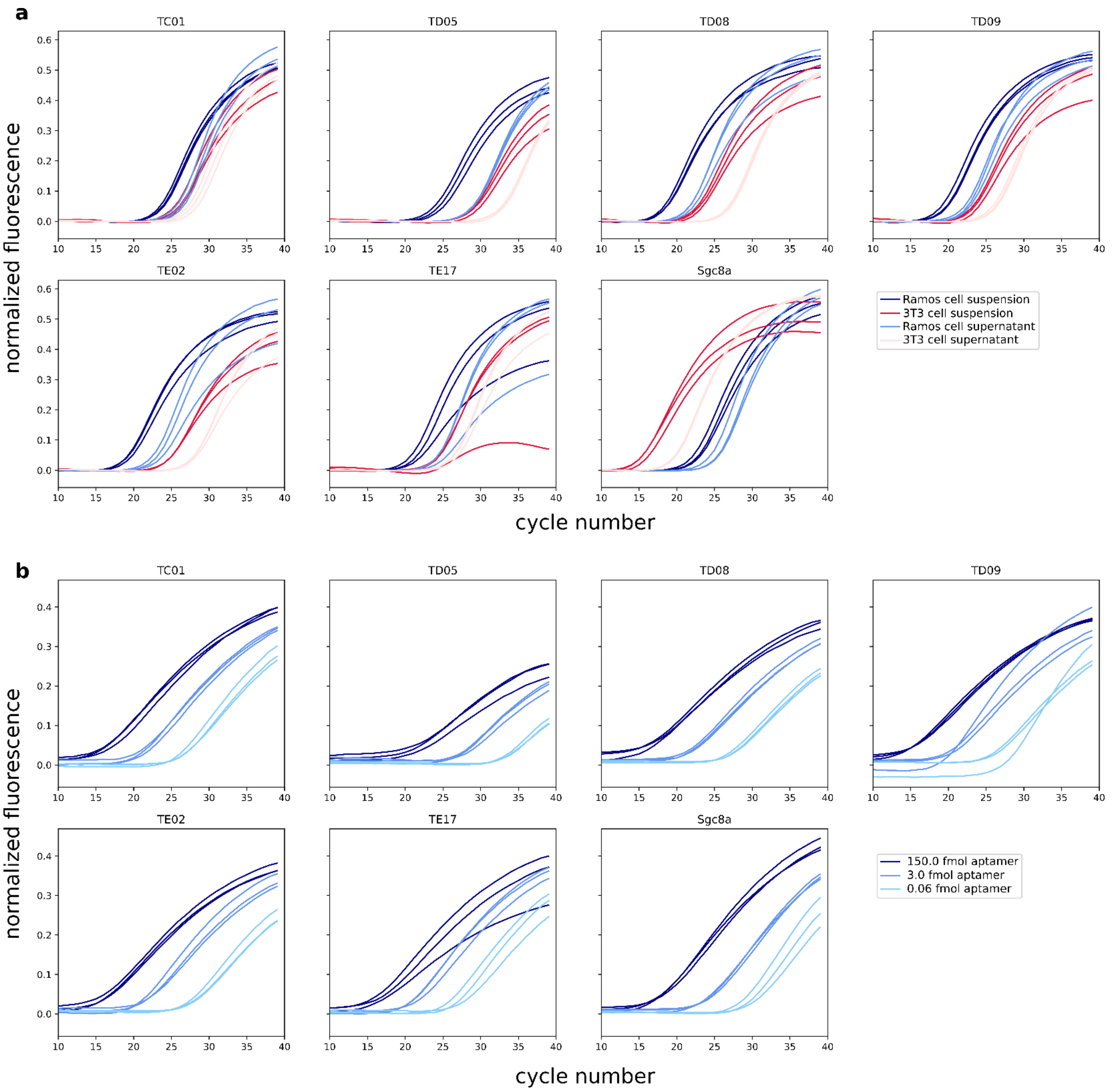

Supplementary Figure 1: qPCR amplification plots of all aptamers modified with a poly-A tail. **a** Association of each aptamer with the two cell lines, Ramos and 3T3 is tested. For each aptamer and both cultures either about 4200 cells in suspension 7 µl or as background control 7 µl of supernatant are used as template material. Each curve represents a technical replicate. **b** Corresponding standard curves for each aptamer and associate primer pair is given. In each panel curves represent three independent dilution series.

Supplementary Table 1: Aptamer sequences used in this study. The unique sequence of each aptamer is given in bold face

Name	Sequence
TC01	AAGGAGCAGCGTGGAGGATA **ACCAAACACAGATGCAACCTGACTTCTAACGTCATTTGGTG** TTAGGGTGTGTCGTCGTGGT BAAAAAAAAAAAAAAAAAAAAAA
TD05	AAGGAGCAGCGTGGAGGATA **AACACCGTGGAGGATAGTTCGGTGGCTGTTCAGGGTCTCCTCCCGGTG** TTAGGGTGTGTCGTCGTGGT BAAAAAAAAAAAAAAAAAAAAAA
TD08	AAGGAGCAGCGTGGAGGATA **CTCTAATTGCCGTATAAGGTCAGGGGGTTGGTTGGTTCCTAGTGC** TTAGGGTGTGTCGTCGTGGT BAAAAAAAAAAAAAAAAAAAAAA
TD09	AAGGAGCAGCGTGGAGGATA **GCTGTCATCGGCTTATGTGCTTTCCAGGGTCCGTGCTGTCCGTTG** TTAGGGTGTGTCGTCGTGGT BAAAAAAAAAAAAAAAAAAAAAA
TE02	AAGGAGCAGCGTGGAGGATA **TAGGCAGTGGTTTGACGTCCGCATGTTGGGAATAGCCACGCCT** TTAGGGTGTGTCGTCGTGGT BAAAAAAAAAAAAAAAAAAAAAA
TE17	AAGGAGCAGCGTGGAGGATA **CAGCTACGCAATACAAAACTCCGAACACCTGCTTCTGACTGGGTGCTG** TTAGGGTGTGTCGTCGTGGT BAAAAAAAAAAAAAAAAAAAAAA
sgc8a	ATACCAGCTTATTCAATT **ACACTTAGAGTTCTAACTGCTGCGCCGCCGGGAAAATACTGTACGGTT** AGATAGTAAGTGC BAAAAAAAAAAAAAAAAAAAAAA
sgc3b	ATACCAGCTTATTCAATT **CCTGTGGGAAGGCTATAGAGGGGCCAGTCTATGAATA** BAAAAAAAAAAAAAAAAAAAAAA

Supplementary Table 2: Primer sequences

Primer	Sequence
TC/TD/TE nextera adapter	GTCTCGTGGGCTCGGAAGGAGCAGCGTGGAGGATA
sgc8a nextera adapter	GTCTCGTGGGCTCGGATACCAGCTTATTCAATTACACTTAGAG
sgc3b nextera adapter	GTCTCGTGGGCTCGGATACCAGCTTATTCAATTCCTGTGGG
TC01 qPCR fw	GCGTGGAGGATAACCAAACAC
TD05 qPCR fw	GCGTGGAGGATAAACACCGTG
TD08 qPCR fw	GCGTGGAGGATACTCTAATTGCC
TD09 qPCR fw	GCGTGGAGGATAGCTGTCATC
TE02 qPCR fw	GCGTGGAGGATATAGGCAGTG
TE17 qPCR fw	GCGTGGAGGATACAGCTACG
sgc3b qPCR fw	ATACCAGCTTATTCAATTCCTGTGGG
sgc8a qPCR fw	ACCAGCTTATTCAATTACACTTAGAG
TC/TD/TE qPCR rv	ACCACGACGACACACCC
sgc3b qPCR rv	GAGGGGCCAGTCTATGAATA
sgc8a qPCR rv	GCACTTACTATCTAACCGTACAG
DropSeq smart pcr	AAGCAGTGGTATCAACGCAGAGT
DropSeq TSO	AAGCAGTGGTATCAACGCAGAGTGAATrGrGrG
